# **Longer hospital stay but similar one-year mortality and readmission after combined proximal humerus and proximal femur fragility fractures: a Propensity-Score–Matched Cohort Study**

**DOI:** 10.1007/s00402-026-06474-2

**Published:** 2026-09-16

**Authors:** Stephen Murphy, Clara Kellett, Dylan Roberts, Clara McGurk, Emer Ahern, Padhraig O’Loughlin

**Affiliations:** 1 Cork University Hospital, Cork, Ireland; 2 Royal College of Surgeons in Ireland, Dublin, Ireland; 3 University College Cork, Cork, Ireland

**Keywords:** Fragility fracture, Proximal humerus, Proximal femur, Multidisciplinary team, Osteoporosis

## Abstract

**Background:**

Fragility Proximal Humerus (PH) fractures are common and often associated with significant morbidity and mortality. Unlike Proximal Femur (PF) fractures, PH fractures are often omitted from established orthogeriatric multidisciplinary team (MDT) pathways. Combined Proximal Humerus and Proximal Femur fractures (PH&PF) are rare. Combined injuries would be expected to result in worse outcomes compared to isolated PH fractures due to greater injury burden. This study assessed outcomes between isolated PH and combined PH&PF fractures, exploring whether differences in care pathways may contribute to outcome.

**Methods:**

A retrospective propensity score-matched cohort study was performed over a 2-year period identifying patients sustaining isolated fragility PH fractures and over a 5-year period identifying patients sustaining combined PH&PF fractures secondary to low-energy trauma. Propensity score matching was performed using age, sex, comorbidity burden (as defined by Charlson Comorbidity Index (CCI)), mobility status, cognitive impairment and fracture pattern. Primary outcome was 1-year mortality. Secondary outcomes included 1-year readmission and length of stay (LoS). Mortality was analysed using Firth logistic regression, readmission using clustered logistic regression, and LoS using Gamma regression with cluster-robust standard errors.

**Results:**

Sixty-five patients were included following matching (42 PH, 23 PH&PF). Excellent covariate balance was achieved across all matched variables (all absolute standardised mean differences < 0.10). One-year mortality was 7.1% in the PH group and 13.0% in the PH&PF group (OR 1.93, 95% CI 0.38–9.88; *p* = 0.417). Readmission occurred in 33.3% and 26.1% of patients, respectively (OR 0.71, 95% CI 0.17–2.92; *p* = 0.615). Median hospital LoS was 4 days (IQR 0-18.3) for PH fractures and 17 days (IQR 10.5–44.5) for PH&PF fractures. Gamma regression estimated a two-fold increase in hospital LoS among patients with concomitant PH&PF fractures (length-of-stay ratio 2.03, 95% CI 0.98–4.22; *p* = 0.057). Sensitivity analyses incorporating calendar year yielded similar findings.

**Conclusions:**

Despite a greater injury burden, patients with combined PH&PF fractures demonstrated equivocal 1-year mortality and readmission rates versus those with isolated PH fractures. These findings raise the possibility that access to established orthogeriatric MDT pathways may contribute to outcomes and support further prospective investigation into dedicated multidisciplinary care models for fragility PH fractures.

## Introduction

Fragility fractures are defined as a fracture resulting from mechanical forces that would not ordinarily cause fracture, such as low-level (low energy) trauma. Fragility fractures are common, and their incidence is expected to increase with the aging population in Ireland [1]. They are also associated with significant morbidity and mortality [2]. Fractures of the Proximal Femur (PF) have been extensively studied in the literature, primarily due to the traditionally high rates of mortality and morbidity. Initiatives such as the Irish Hip Fracture Database have improved outcomes for these patients, however, 1 year mortality remains relatively high at about 20% [3–5].

Unlike fractures of the PF, fragility Proximal Humerus (PH) fractures have not been as extensively studied. Several papers have addressed the question of surgical versus conservative treatment for these injuries in the older population. Overwhelmingly, conservative management has been shown to produce functional outcomes comparable with surgical management while resulting in fewer complications [6–8]. However, the impact of multidisciplinary team (MDT) involvement, well established in improving outcomes in PF fracture care, has not been assessed in this cohort.

Combined Proximal Humerus and Proximal Femur Fracture (PH&PF) are rare, occurring in approximately 1–2% of PF fractures [9–12].

As both injuries are individually associated with significant morbidity and mortality, it would be expected that patients sustaining PH&PF fractures would have a poorer prognosis when compared with those sustaining isolated fractures. However, we hypothesised that patients sustaining combined PH&PF, and therefore receiving multidisciplinary input, through the established orthogeriatric pathways, may experience improved outcomes and a reduction in adverse events when compared with patients sustaining isolated PH injuries.

To investigate this question, we conducted a retrospective case cohort review of all isolated PH and combined PH&PF in our unit to assess whether there was a significant difference in 1-year mortality and readmission rates between these two cohorts.

## Methods

### Study design

A retrospective review of the orthopaedic electronic referral list was conducted to identify all patients with radiologically confirmed isolated PH fractures secondary to low energy trauma (i.e. fall from standing height) over a 2-year period from January 2022 to January 2024. To identify patients with combined PH&PF fragility fractures, the search period was extended to five and a half years, from June 2019 to December 2024, to maximise case capture of this rare, combined injury. June 2019 was selected to coincide with the introduction of the orthogeriatric service to our institution, allowing identification of all eligible patients managed through this pathway. To the authors’ knowledge, there were no significant changes to referral practice, orthogeriatric and MDT services, availability of rehabilitation, discharge policies or operative treatment during this period.

No age restriction was enforced. Instead, fragility fractures were defined solely on mechanism of injury, defined as low-energy trauma resulting in fracture. The study aimed to compare outcomes between patients with isolated PH fragility fractures and those with combined PH&PF fragility fractures. Primary and secondary outcomes, matching variables and sensitivity analyses were prespecified prior to analysis.

Patients identified had their electronic medical and radiological records screened against inclusion and exclusion criteria (Table 1.) All included patients had a minimum of one year of follow-up.

**Table 1:** Inclusion and exclusion criteria

Inclusion criteria	Exclusion criteria
Radiologically confirmed PH and PH&PF fractures	Fracture dislocations of PH
Fractures secondary to low energy trauma	Fractures secondary to high energy trauma
	Pathological fractures

### Data collection

**Fig. 1:**
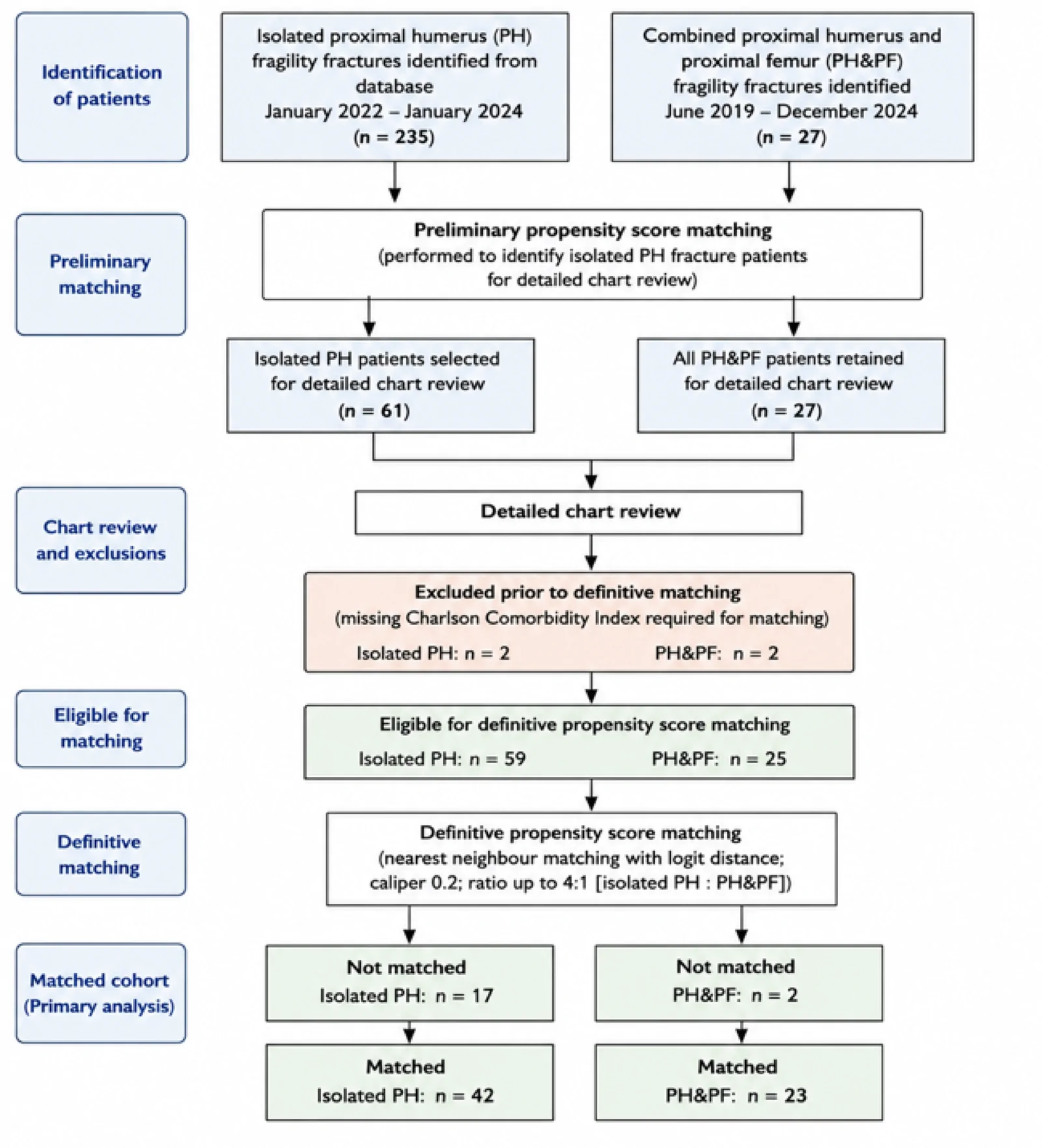
Flow diagram of patient identification, chart review, exclusions, and propensity score matching. *PH* proximal humerus, *PH&PF* proximal humerus and proximal femur

Demographic and clinical details from identified patients were recorded, including age, sex, comorbidity burden, quantified using the validated Charlson Comorbidity Index (CCI), baseline mobility, cognitive impairment, nursing home resident, and Neer classification. Additional variables included treatment modality, admission status, LoS and 1-year readmission and mortality rates. Semi-elective staged readmission for patients treated with surgical fixation but initially discharged was not included within the 1-year readmission group. To facilitate detailed chart review, which was required to obtain clinical variables not routinely available within the institutional fracture database, a preliminary propensity score matching procedure was undertaken to identify isolated PH fracture patients for detailed data collection. This preliminary matching used age, sex, Neer classification, baseline mobility, cognitive impairment, diabetes, cardiac disease, COPD, CKD and active cancer history as matching variables. Nearest-neighbour propensity score matching with a logit distance, calliper width of 0.2 and a maximum matching ratio of 4:1 was used. All patients with combined PH&PF fractures were retained. The participant selection process, exclusions and propensity score matching are illustrated in Fig. 1.

**Fig. 2:**
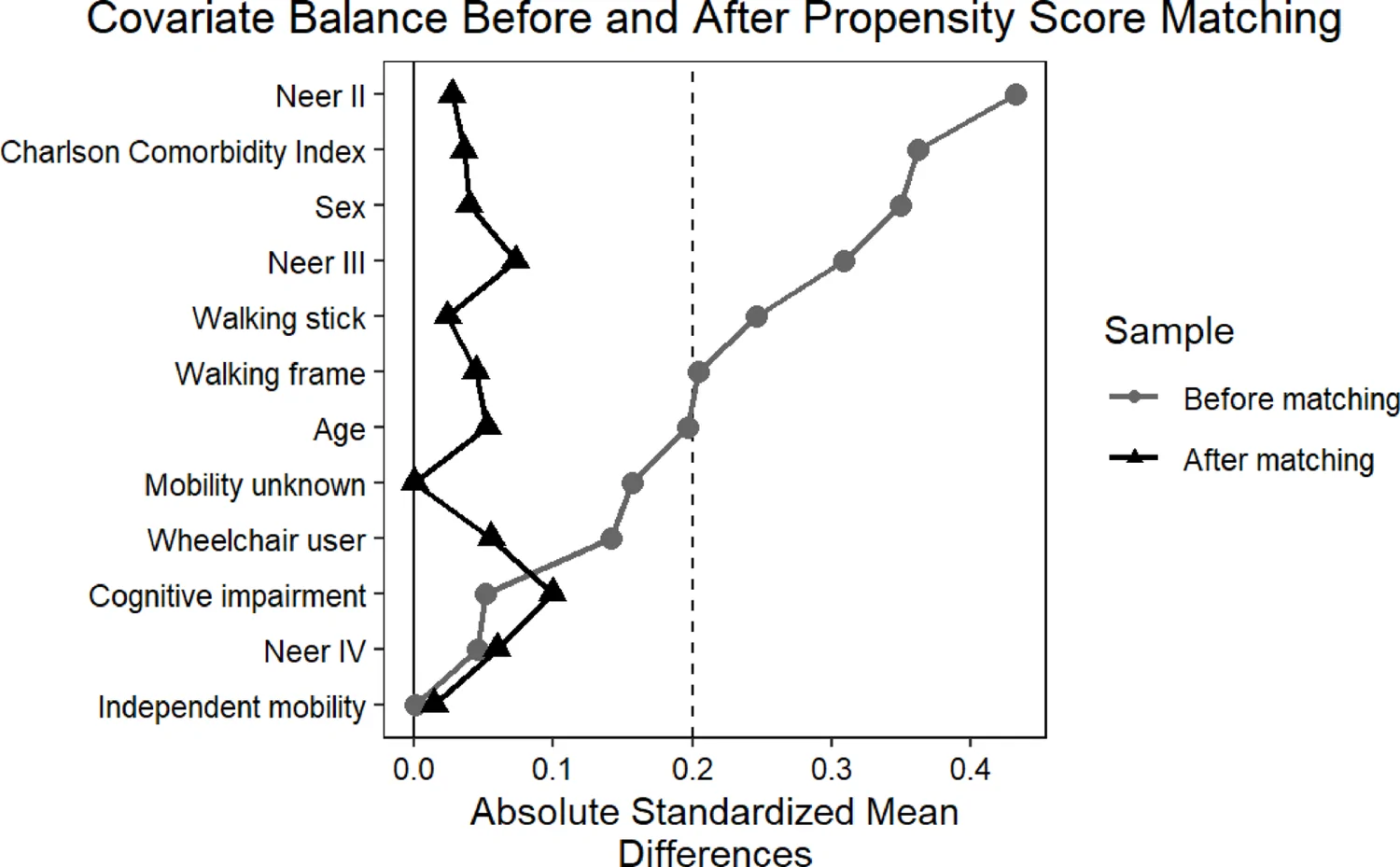
Love plot demonstrating standardised mean differences before and after propensity score matching

Following detailed chart review, patients with missing Charlson Comorbidity Index data required for propensity score matching were excluded. Complete-case analysis was performed. No imputation was undertaken. Definitive propensity score matching was then performed for the primary analysis using age, sex, CCI, mobility, cognition and Neer fracture classification. Nearest-neighbour propensity score matching with a logit distance, calliper width of 0.2 and a maximum matching ratio of 4:1 (PH: PH&PF) was performed using the MatchIt package (version 4.7.2) in R. Covariate balance before and after matching was assessed using standardised mean differences and visual inspection of a Love Plot (Fig. 2). An absolute standardised mean difference < 0.20 considered acceptable. Visual inspection of propensity score distributions demonstrated adequate overlap between groups before matching, with no observations discarded because of lack of common support.

### Outcome measures

The primary outcome was 1-year mortality. Secondary outcomes were hospital readmission within 1-year and hospital LoS. LoS was analysed only among admitted patients as patients discharged directly from the emergency department do not have an inpatient hospital stay. Mortality and readmission data were obtained from the HSE South electronic healthcare record. Events occurring outside the HSE South hospital network could not be captured. Patients who died before one year were classified according to whether a readmission had occurred before death.

### Statistical analysis

Continuous variables were summarised using means and standard deviations or medians and interquartile ranges (IQR), as appropriate. Categorical variables were summarised using counts and percentages.

Hospital admission was summarised descriptively. Because LoS was positively skewed, it was analysed using a generalized linear model with Gamma distribution and log link. Cluster-robust standard errors were used to account for the matched-set structure.

Readmission was analysed using logistic regression with cluster-robust standard errors to account for the matched-set structure. Because only six deaths occurred during follow-up, one-year mortality was analysed using Firth penalised logistic regression to reduce sparse-data bias.

Covariate balance following propensity score matching was assessed using standardised mean differences. All covariates demonstrated excellent balance after matching, with absolute standardised mean differences < 0.10. Although covariate balance after matching was excellent, prespecified adjusted analyses were performed to assess the robustness of the findings.

A prespecified sensitivity analysis incorporating calendar year into the propensity score model was performed to assess the potential influence of differing recruitment periods between cohorts.

All statistical analyses were performed using R version 4.5.2 (R Foundation for Statistical Computing, Vienna, Austria). Propensity score matching was performed using the MatchIt package (version 4.7.2). Cluster-robust variance estimation was performed using clubSandwich (version 0.7.0), and Firth penalised logistic regression was performed using logistf (version 1.26.1). A two-sided P value < 0.05 was considered statistically significant.

## Results

Following propensity score matching, the final matched cohort comprised 65 patients: 42 with isolated PH fractures and 23 with concomitant PH&PF fractures. Baseline characteristics of the matched cohort are summarised in Table 2.

Patients with concomitant PH&PF fractures required hospital admission in all cases. 71.4% of isolated PH fractures required admission. Conservative treatment predominated in both groups (78.3% vs. 76.2%). One-year mortality occurred in three patients in each cohort, while readmission occurred in 14 (33.3%) PH patients and 6 (26.1%) PH&PF patients.

Following propensity score matching, excellent covariate balance was achieved across all matched variables. Absolute standardised mean differences were < 0.10 for all baseline covariates (Table 3; Fig. 2).

**Table 2:** Baseline characteristics after propensity score matching

Baseline characteristics after propensity score matching			
Variable	PH (n = 42)	PH&PF (n = 23)	Absolute SMD
Age, Median (IQR)	79 (74, 87)	79 (73, 86)	0.053
Male sex, n (%)	10 (23.8%)	4 (17.4%)	0.015
CCI, median (IQR)	6 (4, 8)	5 (3, 8)	0.036
*Baseline mobility, n (%)*			0.015
Independent	24 (57.1%)	14 (60.9%)	
Walking Stick	8 (19.0%)	5 (21.7%)	
Walking Frame	8 (19.0%)	3 (13.0%)	
Wheelchair	2 (4.8%)	1 (4.3%)	
Cognitive impairment, n (%)	9 (21.4%)	4 (17.4%)	0.040
*Neer classification, n (%)*			0.036
Two-part	11 (26.2%)	4 (17.4%)	
Three-part	20 (47.6%)	13 (56.5%)	
Four-part	11 (26.2%)	6 (26.1%)	
*Treatment Modality, n (%)*			
Conservative	32 (76.2%)	18 (78.3%)	
ORIF	7 (16.7%)	3 (13.0%)	
TSA	3 (7.1%)	2 (8.7%)	

Continuous variables are presented as median (interquartile range) and categorical variables as number (percentage). Absolute standardised mean differences (SMDs) are reported. For categorical variables with multiple levels, the largest absolute SMD across levels is presented. Absolute SMDs < 0.10 indicate excellent covariate balance after matching

### Mortality

One-year mortality occurred in 3 of 42 patients (7.1%) with isolated PH fractures and 3 of 23 patients (13.0%) with concomitant PH&PF. Firth logistic regression demonstrated no significant association between fracture group and one-year mortality (OR 1.93, 95% CI 0.38–9.88; *p* = 0.417).

### Readmission

One-year readmission occurred in 14 of 42 patients (33.3%) with isolated PH fractures and 6 of 23 patients (26.1%) with concomitant PH&PF. Clustered logistic regression demonstrated no significant association between fracture group and one-year readmission (OR 0.71, 95% CI 0.17–2.92; *p* = 0.615).

### Length of stay

Median LoS during the index hospital admission was 17 days (interquartile range [IQR] 10.5–44.5) in the PH&PF group versus 4 days (IQR 0-18.25) in the PH group.

Gamma regression with cluster-robust standard errors estimated a two-fold increase in hospital LoS among patients with concomitant PH&PF fractures (length-of-stay ratio 2.03, 95% CI 0.98–4.22; *p* = 0.057).

### Summation

Propensity score matching achieved excellent balance across all measured baseline covariates, with absolute standardised mean differences below 0.10 for every matched variable (Table 2; Fig. 2). Patients with concomitant PH&PF fractures experienced longer hospital stays than patients with isolated PH fractures, although no evidence of a difference was observed in one-year mortality or readmission. Clinical outcomes are summarised in Table 3.

**Table 3:** Clinical outcomes in matched cohorts

Clinical outcomes in matched cohorts				
Outcome	PH (n = 42)	PH&PF (n = 23)	Effect Estimate (95% CI)	p-value
LoS, median (IQR), days	4 (0-18.3)	17 (10.5–44.5)	LoS ratio 2.03 (0.98–4.22)	0.057
1-year mortality, n (%)	3 (7.1%)	3 (13.0%)	OR 1.93 (0.38–9.88)	0.417
1-year readmission, n (%)	14 (33.3%)	6 (26.1%)	OR 0.71 (0.17–2.92)	0.615

Sensitivity analyses incorporating calendar year into the propensity score yielded similar findings. No significant differences were observed for one-year mortality (OR 2.76, 95% CI 0.52–14.74; *p* = 0.223) or readmission (OR 0.98, 95% CI 0.21–4.49; *p* = 0.980). The association between concomitant PH&PF fractures and prolonged hospital stay persisted and was more pronounced (length-of-stay ratio 3.98, 95% CI 1.64–9.65; *p* = 0.005), supporting the robustness of the primary findings.

## Discussion

This propensity score-matched cohort study compared outcomes following isolated PH fragility fractures versus combined PH&PF fragility fractures. Patients with concomitant PH&PF fractures experienced substantially longer hospital stays. This finding was somewhat expected, as the vast majority of PF fractures undergo surgical fixation upon initial presentation [13]. In contrast, fragility PH fractures are often treated non-surgically, and can often be discharged directly home [7, 14]. Additionally, due to treatment of these combined injuries, rehabilitation may often be prolonged due to factors such as avoiding weight bearing through the affected upper limb [15].

In contrast to discrepancies in LoS, no difference in 1-year readmission rates or mortality were observed. Several previous studies have examined outcomes following isolated PF fracture with combined PF and concurrent osteoporotic fracture, demonstrating higher mortality and morbidity in the combined cohort [11, 16].

A recent study by Shaham et al. examining combined versus PH fractures showed higher mortality and morbidity in the combined cohort, opposing the results found in this study [17]. However, an important methodological difference seen is that cohorts in the study by Shaham et al. were not matched, with the combined cohort demonstrating significantly higher age and Charlson Comorbidity Index (CCI) scores. The authors comment on this and hypothesise that higher CCI and age may result in poorer outcomes for these patients.

One of the notable findings of this study was that patients sustaining combined injuries did not demonstrate significantly worse mortality or readmission rates despite presenting a more clinically vulnerable cohort. It would be expected that sustaining two major fragility fractures would translate to worse outcomes due to the greater physiological insult and impaired mobility and therefore poorer outcomes.

A potential explanation for this may be the contributions of the established orthogeriatric multidisciplinary team and pathways routinely implemented for PF fractures. However, as orthogeriatric involvement, rehabilitation intensity and adherence were not directly measured in this study, this remains a hypothesis rather than a conclusion. Modern PF fracture care has evolved substantially over the previous decades due to evidence demonstrating improved outcomes via coordinated MDT management involving orthopaedic surgeons, gerontological medicine, physiotherapy, occupational therapy and specialist nursing staff. National initiatives such as the Irish Hip Fracture Database have further standardised care and with improved perioperative optimisation, early and aggressive rehabilitation, osteoporosis treatment and falls assessment.

In contrast to this, PH fractures do not routinely receive this level of specialist care. In many centres, the majority of these injuries are managed conservatively with variable access to physiotherapy and geriatric assessment, despite evidence that these fractures represent significant frailty. An alternative explanation for these paradoxical findings may be due to difference in clinical management rather than injury severity. Patients sustaining PF fractures are routinely admitted to an orthopaedic ward, where they receive not only early surgical fixation, but also intensive post-operative physiotherapy and comprehensive geriatric assessment including falls assessment and bone health. Such thorough assessment and rehabilitation are rarely seen with isolated PH fractures, where patients are often discharged directly home from the ED. Consequently, it is plausible that the differences in care pathways may contribute to the observed outcomes reported. Again, this cannot be determined by this analysis, as factors such as orthogeriatric and MDT input were not directly examined.

It is plausible that patients sustaining combined PH&PF fractures may derive benefit from a more comprehensive orthogeriatric assessment secondary to this mandatory admission and access to orthogeriatric pathways triggered by the PF fracture. As the level of MDT input was not directly assessed and residual imbalance remained following matching, it cannot be concluded that these pathways improved outcomes. The authors acknowledge that, owing to the limitations of the study design and data available, these findings are hypothesis-generating rather than implying a protective effect.

These findings should, however, be interpreted with caution. There are several limitations to this study.

Firstly, the relatively small, matched cohort limited statistical power, particularly for mortality analyses, as reflected by the wide confidence intervals around effect estimates. Propensity score matching achieved excellent balance across measured baseline covariates; however, residual confounding from unmeasured variables cannot be excluded.

Secondly, orthogeriatric input was referred from treatment pathway rather than quantified directly from patients’ charts. Variables such as frequency of review, intensity of rehabilitation and osteoporosis treatment were not available for analysis and consequently, the proposed protective effect remains hypothesis-generating rather than confirmatory.

Thirdly, the retrospective design is inherently flawed at examining the effect of the MDT on patient outcomes. A more robust study would be a prospective study, randomising MDT care and comparing outcomes. This would, however, raise ethical concerns of undertreating a certain cohort, potentially leading to worse outcomes.

Finally, the study cohorts were derived from partially overlapping recruitment periods, introducing the potential for temporal bias related to evolving clinical practice. However, sensitivity analyses incorporating calendar year into the propensity score yielded findings consistent with the primary analyses, suggesting that differences in recruitment period were unlikely to have materially influenced the observed results.

Even with these significant limitations, we believe this study highlights an important and often under-recognised issue. Fragility PH fractures represent a marker of frailty deserving greater attention from the orthogeriatric and MDT disciplines. While operative interventions may not routinely improve functional outcomes, enhanced MDT assessment may still improve broader patient-centred outcomes such as falls prevention, rehabilitation, osteoporosis identification and treatment, and reduction in readmission. Although this study did not directly show that these pathways improved outcomes, it does raise the possibility that this could be a contributing factor. Further prospective studies are needed to assess whether dedicated orthogeriatric and MDT pathways for PH fractures could improve outcomes, similarly to those seen with the establishment of the hip fracture models.

## Data Availability

All data used was generated by the authors and is available upon request.
